# Highly conductive and flexible color filter electrode using multilayer film structure

**DOI:** 10.1038/srep29341

**Published:** 2016-07-04

**Authors:** Jun Hee Han, Dong-Young Kim, Dohong Kim, Kyung Cheol Choi

**Affiliations:** 1 School of Electrical Engineering, KAIST, Daejeon, Republic of Korea

## Abstract

In this paper, a high performance flexible component that serves as a color filter and an electrode simultaneously is suggested. The suggested highly conductive and flexible color filter electrode (CFE) has a multilayer film structure composed of silver (Ag) and tungsten trioxide (WO_3_). The CFE maintained its color filtering capability even when the films were bent on a polyethylene terephthalate (PET) film. Low sheet resistance of the CFE was obtained using WO_3_ as a bridge layer that connects two Ag layers electrically. The sheet resistance was less than 2 Ω/sq. and it was negligibly changed after bending the film, confirming the flexibility of the CFE. The CFE can be easily fabricated using a thermal evaporator and is easily patterned by photolithography or a shadow mask. The proposed CFE has enormous potential for applications involving optical devices including large area devices and flexible devices.

As display technologies such as organic light-emitting diodes and liquid crystal displays are receiving a great deal of attention, and with advances in image sensing technologies, color filters have become important device components. A color filter is a type of band-pass filter and it is normally made of organic dyes or pigments in industrial use. However, such color filters have low chemical stability and color degraded by the influence of ultraviolet and strong light intensities^1^,^2^,^3^. In efforts to solve these problems, many studies on optical color filters have been conducted. Plasmons from metallic nanostructures are used to make optical color filters^4^,^5^,^6^,^7^,^8^,^9^,^10^, and guided mode resonance is one of the ways used to implement optical color filters^11^,^12^,^13^,^14^,^15^. An electrode is another important element of optical devices and electronics. Although indium tin oxide (ITO) is usually used as an electrode in optical devices and electronics, its sheet resistance is dramatically changed after being bent, thus illustrating that ITO is not a suitable electrode for flexible electronics. To produce electrodes for flexible devices, alternative flexible electrodes such as metal nanowires^16^,^17^, graphene^18^,^19^ , and conductive polymers^20^,^21^ have been studied.

In this study, a high performance flexible component that operates as an electrode and a color filter simultaneously has been developed. Compared with previously reported components^22^,^23^,^24^, flexibility is achieved for the first time and the proposed color filter electrode (CFE) has remarkably low sheet resistance of less than 2 Ω/sq. The optical and electrical properties were negligibly changed after a bending test, demonstrating that the CFE can be exploited in optical flexible devices. The low sheet resistance confirms its high conductivity and it shows potential as an electrode for large area optoelectronics. The low sheet resistance is realized by the use of tungsten oxide (WO3) to allow two silver (Ag) films to be electrically connected. The electrical connection of the Ag layers through WO_3_ was analyzed theoretically and experimentally in this paper. The CFE, which is composed of multiple thin films with tungsten trioxide (WO3) and silver (Ag), uses guided mode resonance to realize the color filter, thereby maintaining the advantages of an optical color filter. By varying the thicknesses of the layers, other colors except red, green, and blue (RGB) also can be realized easily. In order to fabricate the CFE, additional modification of existing fabrication systems is not necessary because it can be easily prepared by thermal deposition. Patterning is also easily realized. A photolithography method and a shadow mask, which are commonly used for patterning in industrially, could be applied to the CFE and sub-10 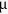
m patterning of the CFE was confirmed experimentally.

## Results and Discussion

**Figure 1:**
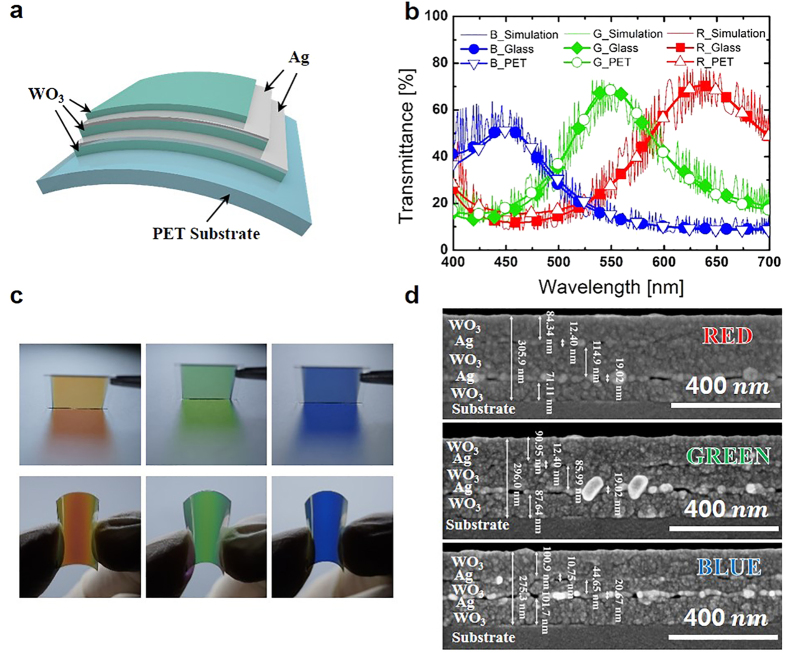
Schematic and characterization of fabricated devices. (**a**) Graphical representation of the conductive and flexible CFE with a multilayer structure consisting of silver and tungsten trioxide (WO_3_) on a polyethylene terephthalate (PET) substrate. (**b**) Transmittance of red, green, and blue CFE with optimized WO_3_ thickness. The solid lines with red, green, and blue are the calculated data. Red colored squares, green colored diamonds, and blue colored circles are experimental data of the red, green, and blue CFEs on a glass substrate, respectively. Red empty triangles, green empty circles, and blue empty inverted triangles are experimental data of the red, green, and blue CFEs on a PET substrate, respectively. (**c**) Images of the realized CFEs. The first column shows the red, green, and blue CFEs on glass substrates, from left to right. The second column shows the red, green, and blue CFEs on PET substrates, from left to right. (**d**) Scanning electron microscope (SEM) images of red, green, and blue CFEs in cross section view. The thickness of each layer is indicated on the SEM images.

Figure 1(a) schematically illustrates the CFE suggested in this paper. As shown in the figure, the structure is composed of a multilayer composed of Ag and WO_3_. The thickness of the bottom Ag layer was 20 nm and that of the top Ag layer was 10 nm. Although the thicknesses of the Ag layers were fixed in this study, the sheet resistance and transmittance of CFE could be altered by adopting different Ag thicknesses (Supplementary Figure 1, Supplementary Figure 2, Supplementary Table 1). The filtered color can be altered by varying the thickness of the dielectric layer located between two metal layers because of the cavity resonance^14^,^25^,^26^,^27^,^28^. Moreover, higher transmittance can be obtained by optimizing the layer thickness of the outer layer, which is located at the outside of the metal layer because this layer provides anti-reflection^15^,^22^,^29^. Furthermore, the outer layer reduces the difference of the admittance between the CFE and the incident medium when it has an optimized thickness^22^, reducing the reflection and improving the transmittance. Therefore, a 5-layer structure was adopted in this study to achieve saturation of filtered color. In order to optimize the thicknesses of the WO_3_ layers for the RGB color filter, MATLAB was used to calculate the characteristic matrix (Supplementary Figure 3, Supplementary Table 2)^30^. The refractive indices of Ag and WO_3_ were measured experimentally (Supplementary Figure 4). The optimized layer thicknesses obtained from the calculation for the RGB CFE are shown in Table 1.

Figure 1(b) shows the calculated and experimental transmittance results for the RGB CFEs with the optimized layer thicknesses. As Fig. 1(b) shows, the transmittance data of the RGB CFEs fabricated on glass and PET had similar transmittance to that obtained by calculation. Fig. 1(c) shows the optimized RGB CFEs fabricated on glass (first column) and polyethylene terephthalate (PET) film (second column). When the CFE was forcibly bent, its optical properties were maintained (Supplementary Video 1). When color coordinates of the RGB CFEs were calculated using transmittance data by the color matching function and plotted on the CIE 1931, the points are located on the red, green, and blue regions (Supplementary Figure 5).

Figure 1(d) shows scanning electron microscope (SEM) images of RGB CFE cross-sectional views. The indicated thicknesses on the SEM images were measured by the software of the SEM system and redrawn for clear visibility. The thicknesses of each film observed from the SEM image were similar to the values reported in Table 1. Other CFEs that have different color aside from RGB could be made with different thicknesses of the WO_3_ film (Supplementary Figure 6, Supplementary Table 3).

**Figure 2:**
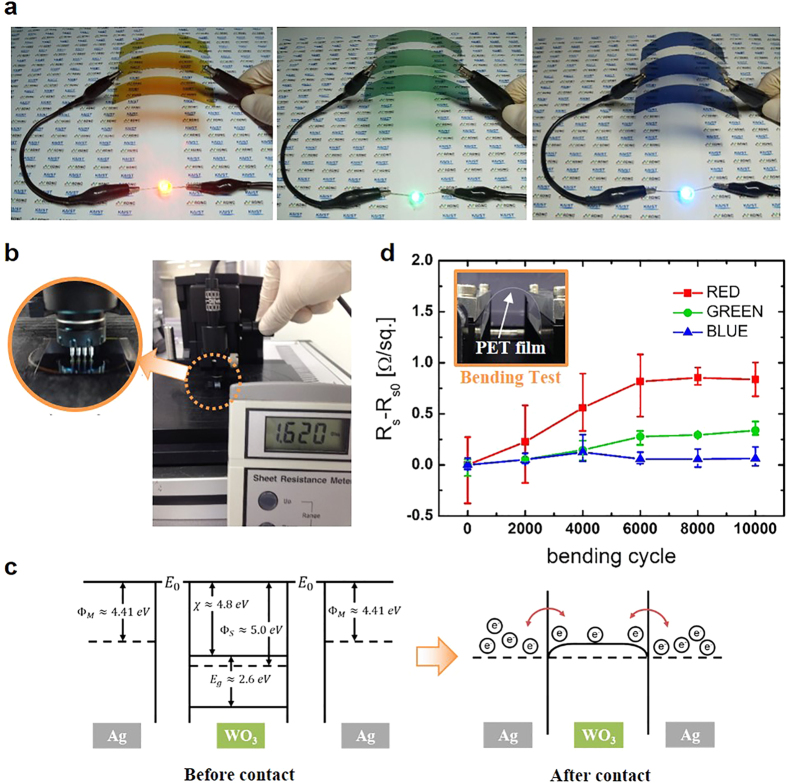
Electrical and mechanical properties of CFEs. **(a)** Images of large sized red, green, and blue CFEs working as a part of an electrical circuit, from left to right. (**b)** Sheet resistance measurement of CFE. (**c)** Schematic diagrams of the energy band structure of WO_3_ and Ag metal. (**d)** Sheet resistance changes after 10 mm radius bending test.

One of the key advantages of the suggested CFE is that it can be easily fabricated. As the Ag and WO_3_ films could be deposited by a thermal evaporation method, the CFE can be made in situ inside a thermal vacuum chamber. Also, the device can be enlarged to as much as the permissible range of the thermal vacuum chamber. A 70 mm by 70 mm size CFE, which corresponded with the largest size possible in our laboratory, was made on a PET substrate (Fig. 2(a)).

A bending test was conducted to check the changes of transmittance after bending, and it was found that the transmittance was negligibly changed after a 10 mm radius, 10,000 cycle bending test, thus verifying its flexibility (Supplementary Figure 7).

Figure 2(a) shows the conductivity of the RGB CFE by having the light emitting diodes (LED) emit light using the RGB CFE as the circuit line. The sheet resistance was measured by using a 4-probe after the entire CFE structure was fabricated. As presented in Fig. 2(b), the CFE provides 1.62 Ω/sq. sheet resistance (Supplementary Figure 8). The RGB CFEs have almost the same sheet resistances due to having the same Ag layer thicknesses. Considering that the sheet resistance of the CFE is about 1.66 Ω/sq. when a 20 nm thick Ag layer with resistance of 1.99 Ω/sq. and a 10 nm thick Ag layer with resistance of 10.22 Ω/sq. are connected electrically in parallel, the measured sheet resistance,1.62 Ω/sq., verifies that the two Ag layers embedded in the CFE were connected electrically (Supplementary Figure 1, Supplementary Figure 9)^31^. Considering that the sheet resistance of ITO is about 10 Ω/sq.^29^, the sheet resistance of the CFE shows better electrical properties than an ITO electrode. The proposed CFE could be applied to large area displays or electronics, because they require an electrode that has low sheet resistance for lower current loss.

The electrical connection between the two separated Ag layers is attributed to the WO_3_ film placed between the two Ag layers. Figure 2(c) schematically illustrates energy band structures that would be expected before and after the contact of Ag and WO_3_ layers^32^,^33^,^34^. After the layers are brought into contact, the Fermi levels align at equilibrium in the ideal case according to the classic metal-semiconductor contact theory. Because of band bending at the contact interface, barriers to electron flow between the Ag and WO_3_ layers are low, as shown in Fig. 2(c), and the electrons easily flow between the two Ag layers through the WO_3_ film. Supplementary Figure 10 shows experimental results, validating that the WO_3_ layer connects two Ag layers electrically.

The CFE fabricated with large size maintained its conductive property while being subjected to bending (Supplementary Video 2). Figure 2(d) shows the change of the sheet resistance of RGB CFEs after a 10 mm radius bending test with the number of bending cycles. The sheet resistances were changed by less than 1 Ω/sq. after 10,000 bending cycles, showing better performance than an ITO electrode, which cracks when bent^29^,^35^,^36^. This shows the potential of the CFE as a flexible electrode. As the CFEs became thicker, the films suffered more tensile stress even though they were bent with the same bending radius^37^. With greater tensile stress, there is a stronger likelihood of cracks forming inside the device, and accordingly the sheet resistance of the red CFEs changed slightly more than that of the other CFEs. However, the sheet resistance change was saturated because the applied stress to the CFEs appears not to exceed the fatigue strength during the 10,000 bending cycles with a 10 mm bending radius, where the crack size remained unchanged^38^.

**Figure 3:**
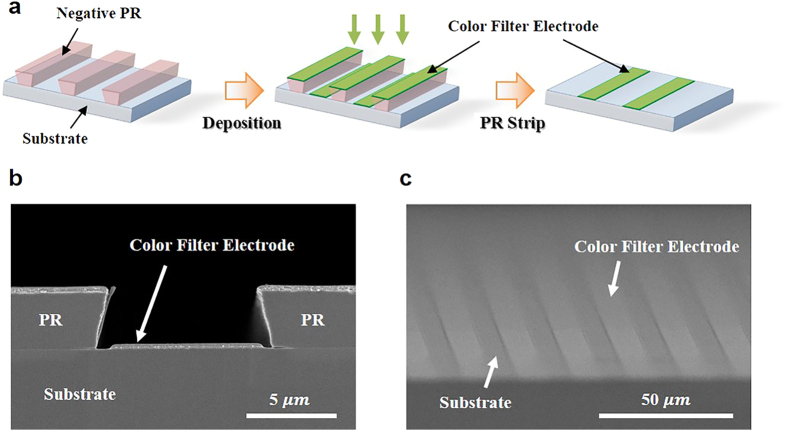
Patterning of the CFE. **(a**) Schematic flow of CFE patterning. (**b**) Scanning electron microscope (SEM) image of a cross section view of deposited CFE on the patterned photoresist. (**c**) SEM image of a tilted view of patterned CFE.

The CFE suggested in this paper could be easily patterned by photolithography because Ag and WO_3_ do not chemically react with acetone, which is used to strip the photoresist (PR). Figure 3(a) schematically illustrates the flow of the CFE patterning process. A negative PR was used as a patterning mask because it has a reversed trapezoidal shape when patterned, as shown in Fig. 3(b). By using the pattering method described in Fig. 3(a), 10 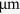-width line patterns of the CFEs were obtained and the sharp edge of the patterned CFE can be confirmed in Fig. 3(b,c). A sub-ten nanometer sized pattern on the CFE might be possible if the scale of the PR pattern width is sufficiently small.

**Figure 4:**
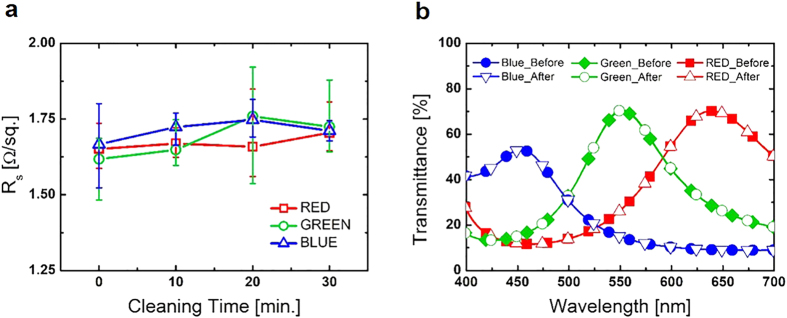
Changes to electrical and optical characteristics of CFEs after acetone cleaning. (**a**) Sheet resistances of red, green, and blue CFE after acetone cleaning with different acetone cleaning time. Red squares indicate the red CFE and green circles indicate the green CFE. Blue triangles indicate the blue CFE. (**b**) Transmittances of red, green, and blue CFEs after 30 min acetone cleaning. Red colored squares, green colored diamonds, and blue colored circles indicate the transmittances of red, green, and blue CFEs before acetone cleaning, respectively. Red open triangles, green open circles, and blue inverted open triangles indicate the transmittances of red, green, and blue CFEs after 30 min acetone cleaning, respectively.

In order to verify that the electrical and optical properties of the CFE were not changed after patterning, the sheet resistances and transmittances of the CFE were evaluated after sonication cleaning with acetone. Figure 4(a) shows that the sheet resistances were not affected by acetone cleaning and Fig. 4(b) shows the CFEs have the same transmittances with and without acetone cleaning. These results confirmed that the electrical and optical properties of the CFE were not changed after the patterning with PR. As Ag and WO_3_ were deposited by thermal evaporation, a shadow mask also could be used for the patterning step. Figure 2(a) shows how a shadow mask works for the patterning. It is seen that three lines of the CFE on a PET substrate were made by the shadow mask. By virtue of the easy patterning methods, it is anticipated that the CFE can be easily applied to various industries.

In summary, a high performance flexible component that serves as an electrode and a color filter simultaneously is fabricated by a thermal evaporation method. The electrical and optical properties of the proposed CFE were maintained after a bending test, thus confirming its flexibility. Also, the pattern of the CFE was easily obtained by photolithography or a shadow mask without altering its electrical and optical characteristics. The CFE suggested in this paper is expected to have a significant impact on the field of display devices, including organic light emitting diodes, liquid crystal displays, and electrophoretic displays. Flexible optoelectronics and large area optoelectronics are another possible field of application of the suggested CFE, as well as CMOS photo sensors and color decoration devices.

## Methods

### Device fabrication

The suggested CFE was manufactured on a cleaned glass substrate and a PET substrate. The thickness of the glass was 700 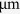 and that of the PET was 125 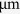. The WO_3_ (1–4 mm pcs 4N, TASCO) and Ag (3–5 mm granule 4N, TASCO) were deposited by thermal evaporation with a vacuum pressure of less than 7 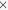 10^−6^ Torr.

### Device characterization

The transmittance spectrum of the CFE at normal incidence was measured by a UV-Vis spectrometer (UV-2550, SHIMADZU). The sheet resistance of the CFE was measured by a 4-point probe (FFP-2400, DASOLENG). A bending test was conducted using a bending machine (Bending system, Custom-made set-up).

### Device patterning

Negative photoresist (NR9-3000PY, FUTURREX INC.) was spin-coated on cleaned glass. In order to coat the photoresist on glass, the sample was accelerated to 2000 RPM for 2 seconds and maintained for 5 seconds, and then accelerated to 4000 RPM for 5 seconds and maintained for 40 seconds. After the spin-coating step, the photoresist was soft-baked at 150 °C for 3 minutes inside an oven. The samples were exposed to ultraviolet light (UV) with a 360 mJ/cm^2^ exposure dose. The photoresist was then baked again at 110 °C inside the oven for 2 minutes. After the baking process, the photoresist was developed for 17 seconds in an AZ300MIF developer. After the photoresist was patterned, multilayers were deposited on the patterned photoresist sample, and then the photoresist was stripped by acetone sonication cleaning for 10 minutes.

### Calculation for the optimized layer thickness

Calculation of the transmittance and optimized layer thickness was performed using MATLAB based on the characteristic matrix method. See supplementary information for more details.

### Material refractive indices

The refractive indices of Ag and WO_2_ were measured experimentally by a spectroscopic ellipsometer (M2000D).

## Additional Information

**How to cite this article**: Han, J. H. *et al*. Highly conductive and flexible color filter electrode using multilayer film structure. *Sci. Rep*. **6**, 29341; doi: 10.1038/srep29341 (2016).

## Supplementary Material

Supplementary Information

Supplementary Movie S1

Supplementary Movie S2

## Figures and Tables

**Table 1:** The optimized layer thicknesses of RGB CFEs.

Color	1^st^ WO_3_	Bottom Ag	2^nd^ WO_3_	Top Ag	3^rd^ WO_3_
RED	72 nm	20 nm	115 nm	10 nm	84 nm
GREEN	87 nm	20 nm	84 nm	10 nm	92 nm
BLUE	100 nm	20 nm	46 nm	10 nm	100 nm
