# Supplementary material for: Highly conductive and flexible color filter electrode using multilayer film structure
Source: Sci Rep. 2016 Jul 4;6:29341. doi: 10.1038/srep29341 (PMC4931689; doi:10.1038/srep29341)
Supplement: Supplementary Information [file srep29341-s1.pdf]

Supplementary Information

Highly conductive and flexible color filter electrode using multilayer film structure

Jun Hee Han[1], Dong-Young Kim[1], Dohong Kim[1], and Kyung Cheol Choi[1,*]

[1]School of Electrical Engineering, KAIST, Daejeon, Republic of Korea

[*]e-mail: kyungcc@kaist.ac.kr

Electrical and optical property changes of CFE by varying the silver (Ag) layer thickness (Supplementary Figure 1, Supplementary Figure 2, Supplementary Table 1).

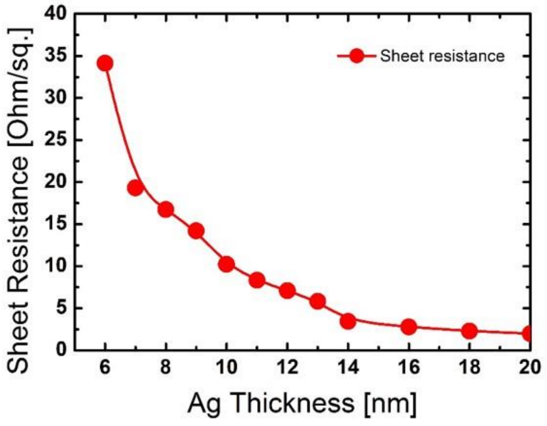

Supplementary Figure 1. Sheet resistances of Ag layers with different thicknesses on WO_3 film.

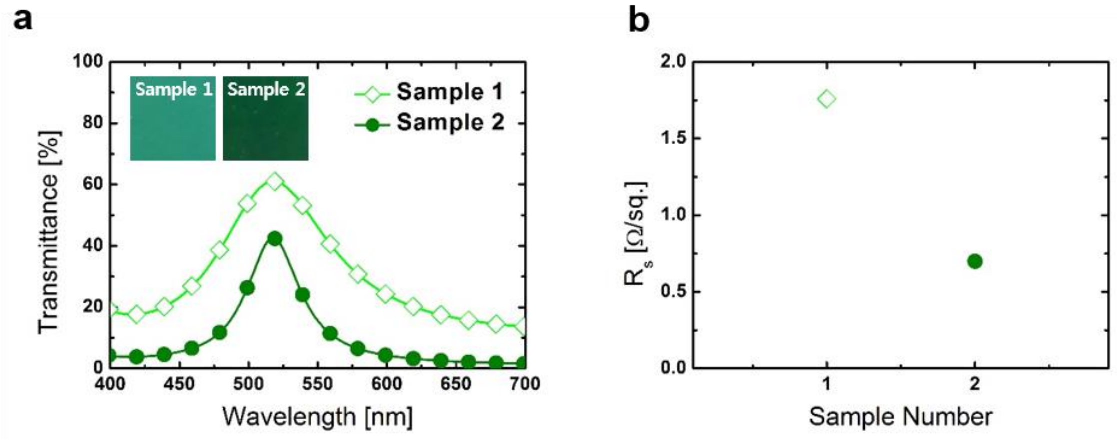

Supplementary Figure 2. Changes to optical and electrical characteristics by varying the thickness of Ag layers. a. Transmittance chances with different thicknesses of Ag layers. The layer thicknesses for both samples are shown in Supplementary Table 1. The inset shows the transmittance color of fabricated samples. **b.** Sheet resistances for both samples.

The sheet resistances were measured by varying the thickness of the Ag layer on tungsten trioxide (WO_3) film. Supplementary Figure 1 shows that the sheet resistances declined as the thickness of the Ag layer was increased. The sheet resistance data of the 7 to 9 nm Ag layer were taken from our previous work [1], and the other sheet resistances were measured experimentally.

Color filter samples having different Ag layer thicknesses were fabricated. As Supplementary Figure 2(a) shows, when the thicknesses of the Ag layers were changed, the optical characteristics also changed. The peak wavelength was negligibly changed even though the thicknesses of the Ag layers were changed because the thickness of the second WO_3 layer mostly determined the peak wavelength [2–6]. Detailed information on the layer thicknesses is presented in Supplementary Table 1.

The sheet resistances of both samples are indicated in Supplementary Figure 2(b). Because sample 2 had greater Ag layer thicknesses, the sheet resistance of this sample was less than that of sample 1, with a value of approximately 0.7 Ω/sq.

Supplementary Table 1. The layer thicknesses of CFEs having different Ag layers thicknesses. The thicknesses of WO_3 layers for both samples were the same.

**Sample**	**1st WO_3**	**Bottom Ag**	**2nd WO_3**	**Top Ag**	**3rd WO_3**
**Sample 1**	87 nm	20 nm	75 nm	10 nm	92 nm
**Sample 2**	87 nm	30 nm	75 nm	30 nm	92 nm

Calculations to obtain the optimized thicknesses of WO_3 film layers for red, green, and blue (RGB) CFEs (Supplementary Figure 3, Supplementary Figure 4, Supplementary Table 2).

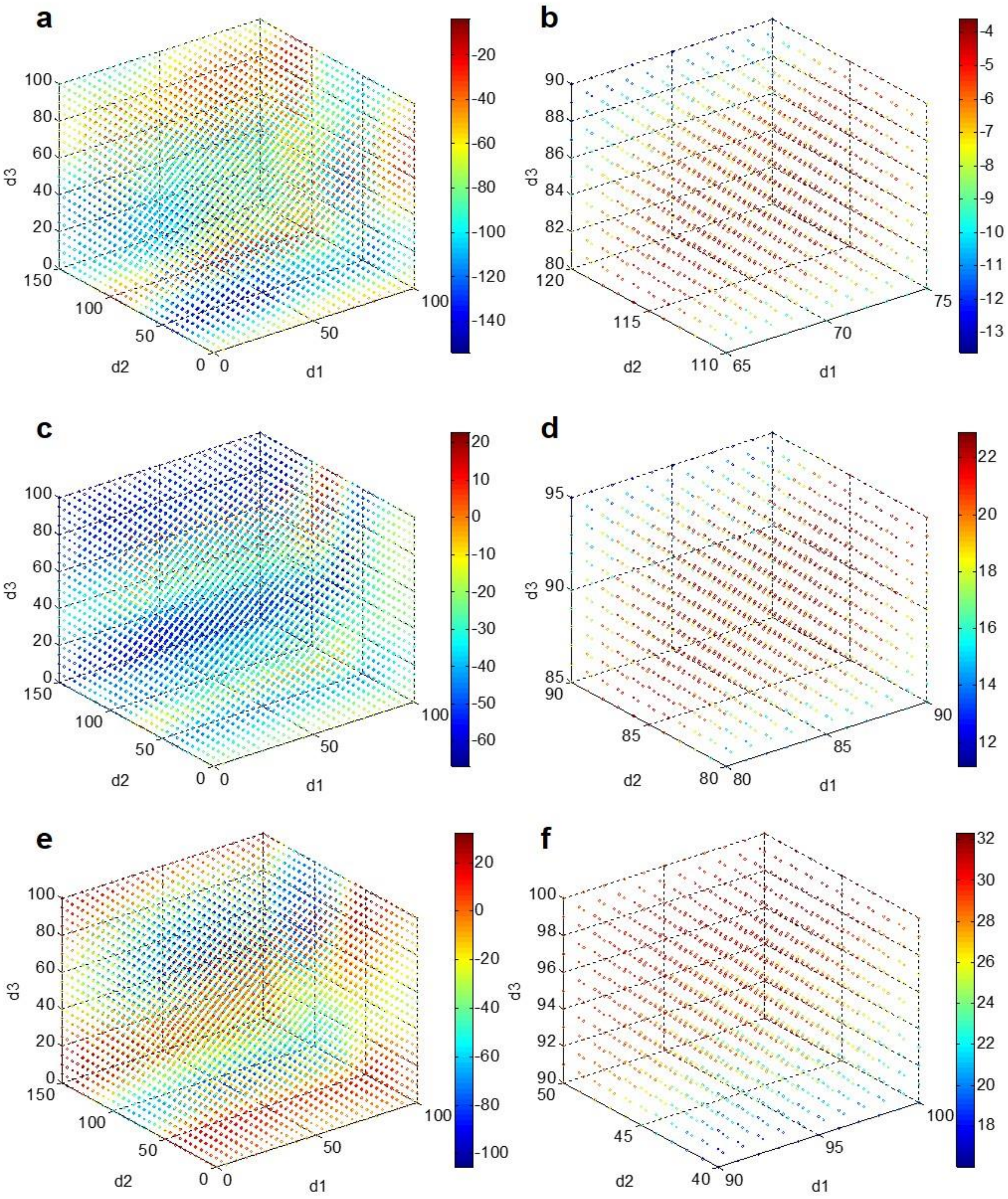

Supplementary Figure 3. Calculation results to obtain the optimized WO_3 films thicknesses for red, green, and blue CFEs. The thicknesses of the bottom, middle, and top WO_3 films were denoted as d1, d2, and d3, respectively. **a and b**. The graphs show the calculated values obtained by subtracting transmittance data at 400 nm, 450 nm, and 550 nm from the transmittance data at 650 nm by varying the thicknesses of the WO_3 layers for the red CFE. **a**. The calculated values are described as colored points at proper positions by varying the WO_3 layers in a range of 0 nm to 100 nm for d1 and d3 and 0 nm to 150 nm for d2 with 5 nm spacing. **b**. The calculated values are described as colored points at proper positions by varying the WO_3 layers in a range of 65 nm to 75 nm for d1, 110 nm to 120 nm for d2, and 80 nm to 90 nm for d3 with 1 nm spacing. **c and d**. The graphs show the calculated values obtained by subtracting transmittance data at 450 nm and 650 nm from the transmittance data at 550 nm by varying the thicknesses of WO_3 layers for the green CFE. **c**. The calculated values are described as colored points at proper positions by varying the WO_3 layers in a range of 0 nm to 100 nm for d1 and d3 and 0 nm to 150 nm for d2 with 5 nm spacing. **d**. The calculated values are described as colored points at proper positions by varying the WO_3 layers in a range of 80 nm to 90 nm for d1 and d2 and 85 nm to 95 nm for d3 with 1 nm spacing. **e and f**. The graphs show the calculated values obtained by subtracting transmittance data of 550 nm and 650 nm from the transmittance data of 450 nm by varying the thicknesses of WO_3 layers for the blue CFE. **e**. The calculated values are described as colored points at proper positions by varying the WO_3 layers in a range of 0 nm to 100 nm for d1 and d3 and 0 nm to 150 nm for d2 with 5 nm spacing. **f**. The calculated values are described as colored points at proper positions by varying the WO_3 layers in a range of 90 nm to 100 nm for d1 and d3 and 40 nm to 50 nm for d2 with 1 nm spacing.

MATLAB was used to calculate the optimized thickness of WO_3 films for RGB CFEs. The calculation was based on the characteristic matrix to observe the transmittance of the multilayer structured CFE [7]. The thicknesses of the bottom Ag layer and the top Ag layer were fixed at 20 nm and 10 nm, respectively. The transmittance data for 400 nm to 700 nm wavelength were calculated by changing the thicknesses of three WO_3 films that compose the color filter, and the transmittance data at the 650 nm, 550 nm, and 450 nm wavelength, which represent red, green, and blue color, respectively, were used to find the optimized WO_3 thicknesses for RGB CFEs.

First, a simulation was conducted with a wide range of WO_3 thickness to observe the tendency of the calculated values. In order to identify the optimized thicknesses of WO_3 layers, the simulation was then conducted again in a specific range of WO_3 thickness referring to the results obtained in the previous simulation. It could be possible to search the optimized thicknesses at once in the wide range of WO_3 layer thicknesses. However, a great deal of time is required to conduct the simulation. For this reason, the calculation is divided into two steps.

For example, in order to decide the thicknesses of WO_3 films for the green color filter, first, we collect the calculated values obtained by subtracting the transmittance data at 450 nm and 650 nm from transmittance data at 550 nm by varying the thicknesses of WO_3 in a range of 0 nm to 100 nm for d1 and d3 and 0 nm to 150 nm for d2 with 5 nm spacing (Supplementary Figure 3(c)). The thicknesses of the bottom, middle, and top WO_3 films are denoted as d1, d2, and d3, respectively. Second, we identify the thicknesses of d1, d2, and d3 that had the highest values among the collected data. Supplementary Table 2 shows d1, d2, and d3 with the highest values among the collected data for RGB CFE. Third, we calculate and collect the values again by subtracting the transmittance data at 450 nm and 650 nm from transmittance data at 550 nm by varying the thicknesses

of WO_3 in a range of ±5 nm relative to the values presented in Supplementary Table 2 with 1 nm spacing (Supplementary Figure 3(d)). Finally, we check the thicknesses of d1, d2, and d3 that had the highest values among the collected data. The final optimized values for the thicknesses of the WO_3 film for the RGB CFE are shown in Table 1 in the main text.

The described calculation steps were conducted for the red and blue color filter in the same manner (Supplementary Figure 3(a), (b), (e), (f)). The transmittance data at 400 nm were subtracted additionally during the calculation for the red CFE because the transmittance at this wavelength in the red CFE tends to rise.

Supplementary Table 2. The layer thicknesses of RGB CFE with the highest value in a range of 0 nm to 100 nm for d1 and d3 and 0 nm to 150 nm for d2.

Color	1st WO_3	Bottom Ag	2nd WO_3	Top Ag	3rd WO_3
**RED**	70 nm	20 nm	115 nm	10 nm	85 nm
**GREEN**	85 nm	20 nm	85 nm	10 nm	90 nm
**BLUE**	100 nm	20 nm	45 nm	10 nm	100 nm

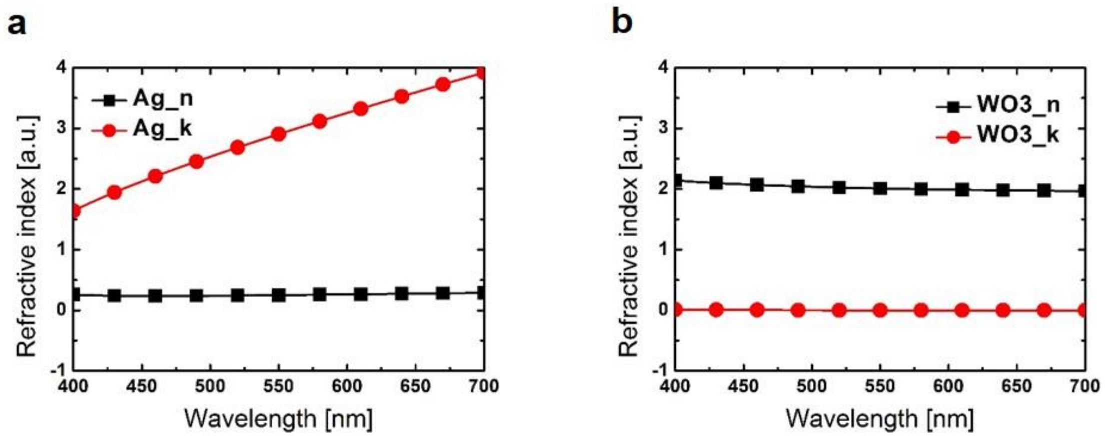

Supplementary Figure 4. Refractive indices of Ag and WO_3.

The refractive indices of Ag and WO_3 were experimentally measured using an ellipsometer (Supplementary Figure 4).

Color coordinates of CFEs on CIE 1931 diagram (Supplementary Figure 5).

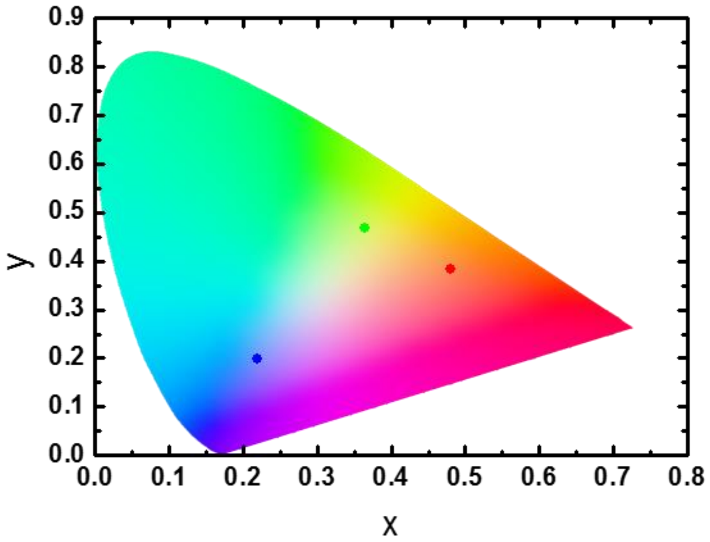

Supplementary Figure 5. CIE 1931 diagram of red, green, and blue CFEs. Red color has a point of (0.48, 0.38) and green color has a point of (0.36, 0.46). Blue color has a point of (0.22, 0.20).

The color coordinates of CFEs were calculated using a color matching function, and they were marked on the CIE 1931 diagram. Red color has a point of (0.48, 0.38) and green color has a point of (0.36, 0.46). Blue color has a point of (0.22, 0.20). As Supplementary Figure 5 shows, color coordinates are located in the red, green, and blue regions.

Various CFEs using suggested multilayer film filter structure (Supplementary Figure 6).

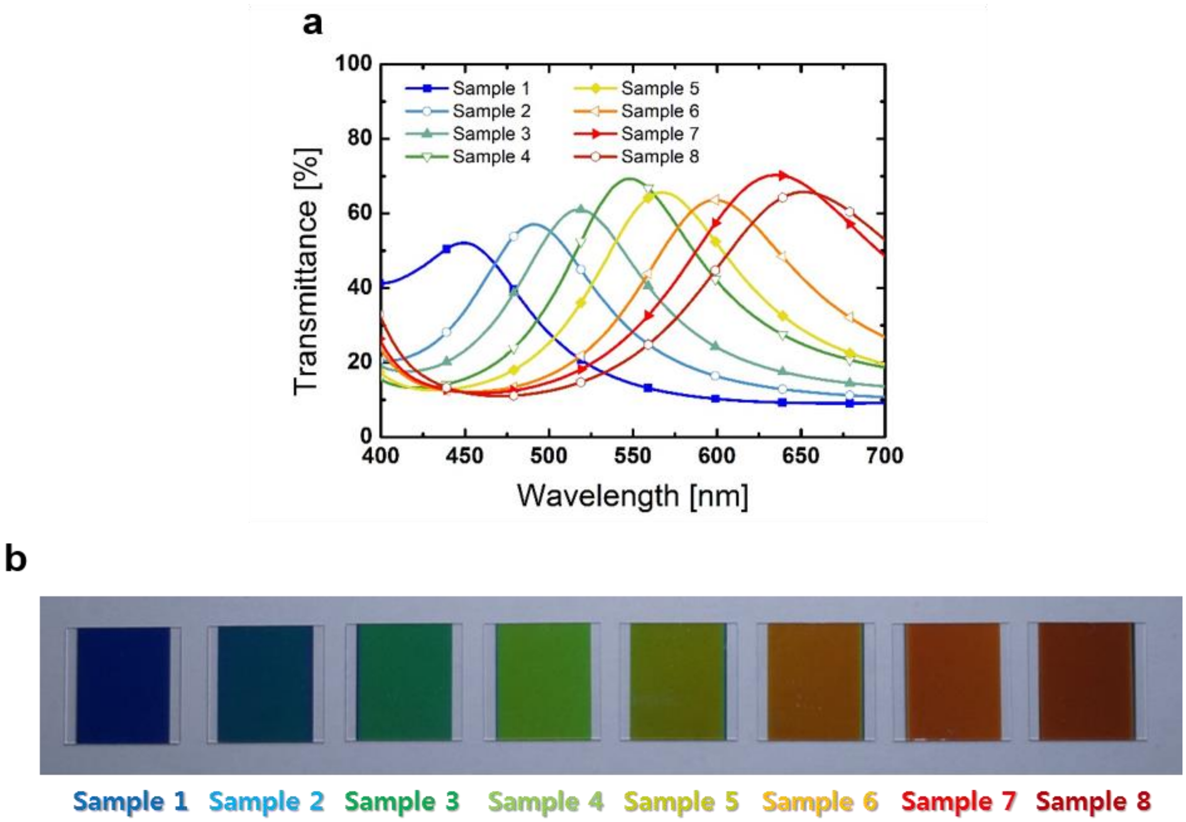

Supplementary Figure 6. Various CFEs. a. Transmittances of various CFEs. **b.** Fabricated CFE samples. The layer thicknesses for various CFEs are shown in Supplementary Table 3.

As supplementary Figure 6 shows, it was possible to prepare CFEs that had various colors by changing the thickness of the WO_3 layer. The thicknesses of the layers for the samples are shown in Supplementary Table 3. The thicknesses for the samples were not optimized, except in samples 1, 4, and 7, which were blue, green, and red, respectively. The sheet resistances of various color filters are roughly 1.7 Ω/sq. because the Ag layers had the same thickness, as described in the main text.

Supplementary Table 3. The layer thicknesses of CFEs having various colors.

Sample	1st WO_3	Bottom Ag	2nd WO_3	Top Ag	3rd WO_3
**Sample 1**	100 nm	20 nm	46 nm	10 nm	100 nm
**Sample 2**	87 nm	20 nm	65 nm	10 nm	92 nm
**Sample 3**	87 nm	20 nm	75 nm	10 nm	92 nm
**Sample 4**	87 nm	20 nm	84 nm	10 nm	92 nm
**Sample 5**	87 nm	20 nm	95 nm	10 nm	92 nm
**Sample 6**	87 nm	20 nm	105 nm	10 nm	92 nm
**Sample 7**	72 nm	20 nm	115 nm	10 nm	84 nm

**Sample 8**	72 nm	20 nm	122 nm	10 nm	84 nm

Optical characteristics of CFE after bending test (Supplementary Figure 7).

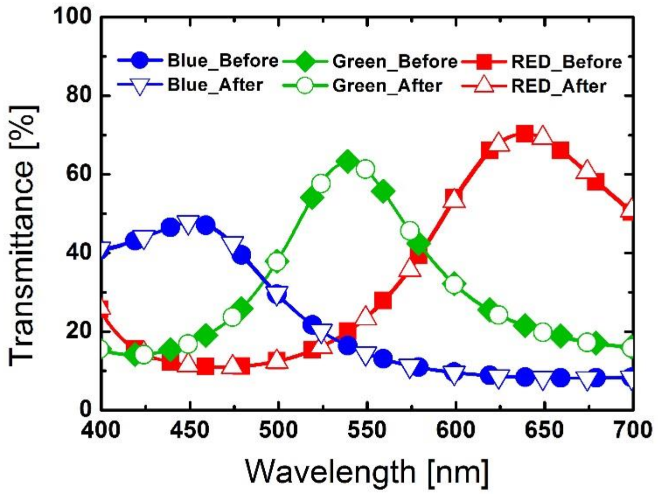

Supplementary Figure 7. Transmittance change of RGB CFEs after 10 mm radius 10,000 cycle bending test. Transmittances of RGB CFEs after 10 mm radius 10,000 cycle bending test. Red colored squares, green colored diamonds, and blue colored circles indicate the transmittances of red, green, and blue CFEs before the bending test, respectively. Red open triangles, green open circles, and blue inverted open triangles indicate the transmittances of red, green, and blue CFEs after the bending test, respectively.

It was shown in the main text that the CFE could be bent on a flexible substrate. For a systematic experiment, a CFE fabricated on a PET substrate was bent for 10,000 cycles with a 10 mm radius and the transmittance was observed. As Supplementary Figure 7 shows, the transmittance was not changed after bending, confirming the flexibility of the CFE.

The sheet resistance measurement system (Supplementary Figure 8).

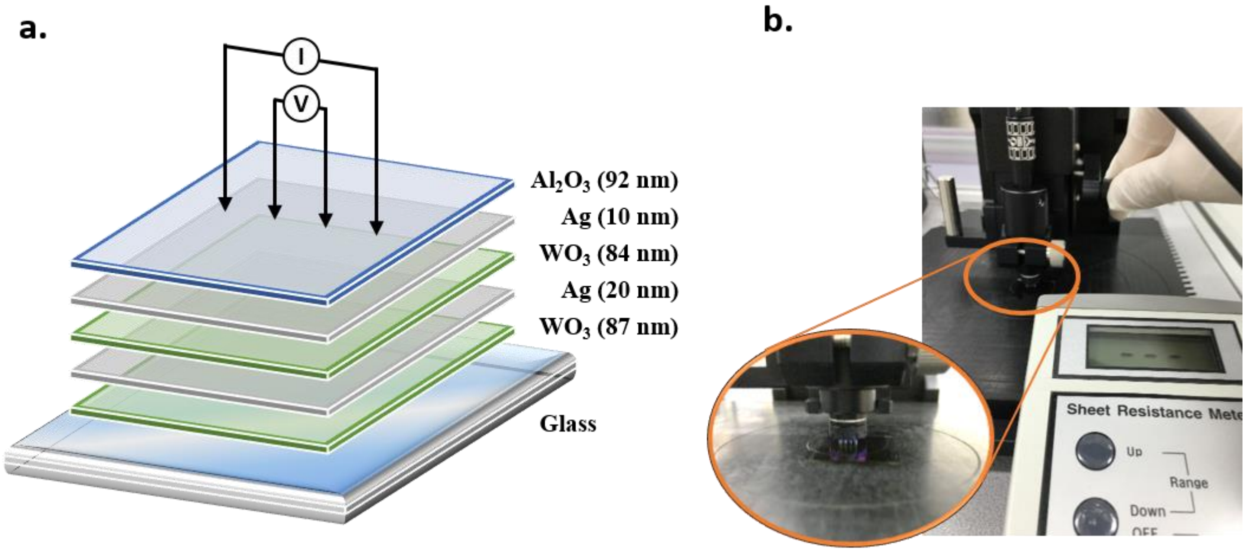

Supplementary Figure 8. Sheet resistance measurement system. a. Schematic of fabricated test device consisting of WO_3, Ag, and Al_2O_3. **b.** Sheet resistance measurement of fabricated test device.

In order to validate that the sheet resistance measurement system did not break through the outer-most layer (WO_3), the device described in Supplementary Figure 8 (a) was fabricated and the sheet resistance was measured. As Supplementary Figure 8 (a) shows, only the oxide material of the outer layer (Al_2O_3) was different from the green CFE and the thicknesses and materials of the other layers were unchanged. Al_2O_3 was deposited with the atomic layer deposition method. When the device was measured, the sheet resistance could not be measured, verifying that the measurement system did not break through the outermost layer (Figure 8 (b)). Therefore it is confirmed that the low sheet resistance of the CFE (less than 2 Ω/sq.) is a reliable figure.

The method to calculate the resistance of Ag / WO_3 / Ag structure (Supplementary Figure 9).

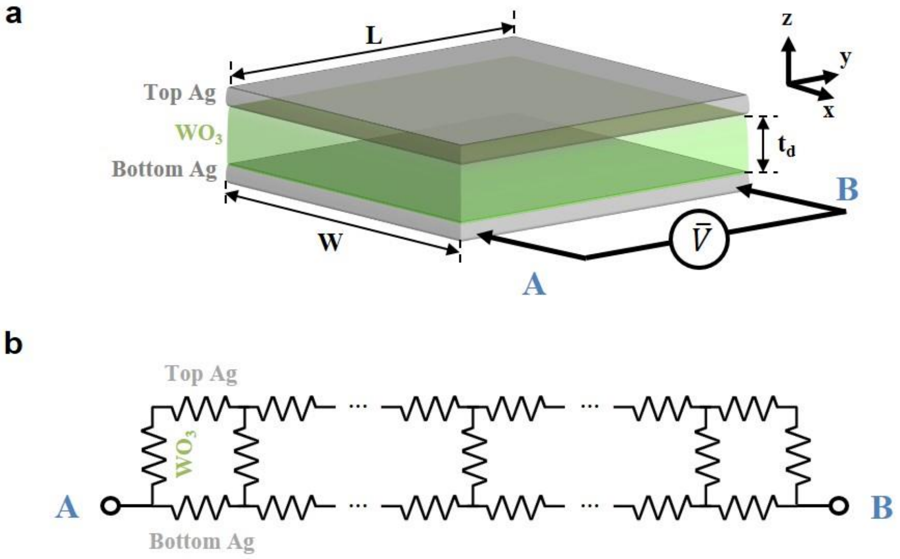

Supplementary Figure 9. Schematic illustration of how the resistance of two connected Ag layers is calculated. a. Schematic of the multilayer consisting of Ag and WO_3. W denotes the width in the x-direction and L denotes the length in the y-direction. t_d indicates the thickness of the WO_3 layer. The schematic shows that the bottom Ag layer was connected to a power supply. **b.** Equivalent resistance circuit model of the multilayers shown in **a.**

Supplementary Figure 9(a) schematically shows the multilayer consisting of Ag and WO_3. The equivalent resistance circuit model is shown in Figure 9(b). The resistance of the Ag / WO_3 / Ag structure can be calculated by solving the equivalent resistance circuit and the result is eq. S1 [8].

$$R_S = \left(\frac{1}{R_{S1}} + \frac{1}{R_{S2}} \right)^{-1} + \left(\frac{R_{S1}\rho_{WO_3}}{\left(1+R_{S2}/R_{S1}\right)^3} \right)^{\frac{1}{2}} \frac{t_d^{\frac{1}{2}}}{L} \qquad \text{eq. S1.}$$

R_{S1} and R_{S2} indicate the sheet resistance of the bottom and top Ag layer, respectively, and ρ_{WO_3} is the resistivity of WO_3. t_d is the thickness of WO_3 and L is the length of the color filter. The second term of eq. S1. converged to zero because the length of the color filter (L) is much greater than the thickness of the WO_3 (t_d). Therefore, the resistance of the Ag / WO_3 / Ag structure can be easily obtained using the first term of eq. S1.

The electrical bridge layer, WO_3. (Supplementary Figure 10).

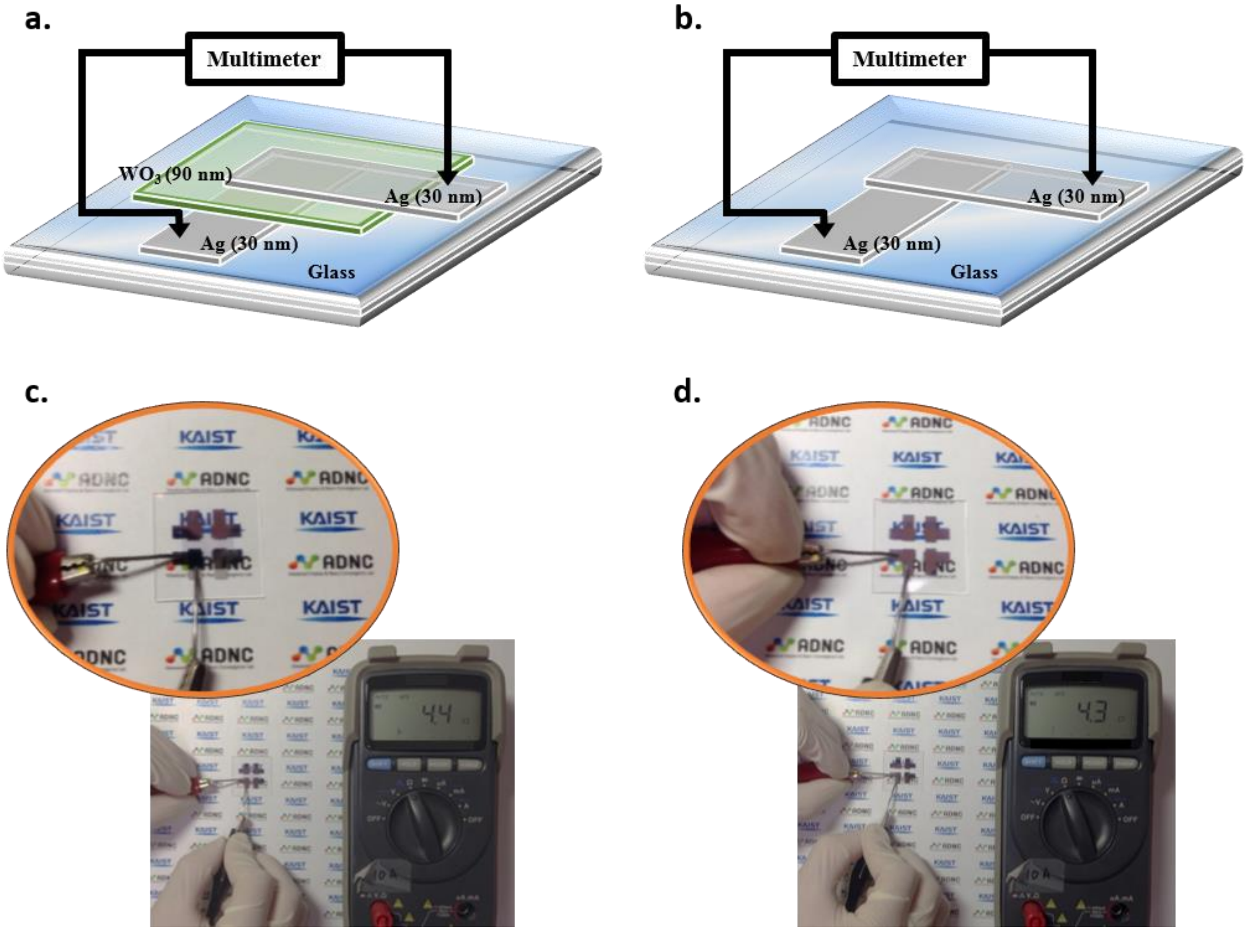

Supplementary Figure 10. Experimental measurement to validate the electrical properties of the WO_3 film between two Ag layers. a. Schematic of the test device consisting of Ag and WO_3. **b.** Schematic of the test device consisting of Ag. **c.** Resistance of the test device consisting of Ag and WO_3. **d.** Resistance of the test device consisting of Ag.

Supplementary Figures 10 (a), (b) present schematics of the test devices and Supplementary Figures 10 (c), (d) respectively show the measured sheet resistances of these devices. As Supplementary Figure 10 (c) shows, two Ag layers were electrically connected even though the WO_3 films covered and physically disconnected the bottom Ag layer from the top Ag layer. It was confirmed from these results that the WO_3 connects two Ag layers electrically.

Supplementary References:

1. Kim, D.-Y., Han, Y. C., Kim, H. C., Jeong, E. G. & Choi, K. C. Highly Transparent and Flexible Organic Light-Emitting Diodes with Structure Optimized for Anode/Cathode Multilayer Electrodes. *Adv. Funct. Mater.* **25,** 7145–7153 (2015).
2. Diest, K., Dionne, J. a., Spain, M. & Atwater, H. a. Tunable color filters based on metal-insulator-metal resonators. *Nano Lett.* **9,** 2579–2583 (2009).
3. Han, J. H., Kim, D.-H. & Choi, K. C. Microcavity effect using nanoparticles to enhance the efficiency of organic light-emitting diodes. *Opt. Express* **23,** 19863 (2015).
4. Kedawat, G., Kumar, P., Vijay, Y. K. & Gupta, B. K. Fabrication of highly efficient resonant structure assisted ultrathin artificially stacked Ag/ZnS/Ag multilayer films for color filter applications. *J. Mater. Chem. C* **3,** 6745–6754 (2015).
5. Minas, G., Ribeiro, J. C., Martins, J. S., Wolffenbuttel, R. F. & Correia, J. H. An array of Fabry-Perot optical-channels for biological fluids analysis. *Sensors Actuators A Phys.* **115,** 362–367 (2004).
6. Yang, C., Shen, W., Zhang, Y., Ye, Z. & Zhang, X. Color-tuning method by filling porous alumina membrane using atomic layer deposition based on metal – dielectric – metal structure. (2013).
7. Born, M. *Principles of Optics: Electromagnitic Theory of Propagation, Interference and Diffraction of Light*. (Cambridge Univ. Press, Cambridge, 2002).
8. Lee, S.-M., Choi, C. S., Choi, K. C. & Lee, H.-C. Low resistive transparent and flexible ZnO/Ag/ZnO/Ag/WO3 electrode for organic light-emitting diodes. *Org. Electron.* **13,** 1654–1659 (2012).

Supplementary Movies

Supplementary Movie 1: The movie version of Figure 1(c) in main text. The bending of blue, green, and red CFEs on the PET substrate is shown in order.

Supplementary Movie 2: The movie version of Figure 2(a) in main text. The LED lights are emitting while the RGB CFEs are being bent. The blue, green, and red CFEs are presented in order.
